# Rev-dependent lentiviral expression vector

**DOI:** 10.1186/1742-4690-4-12

**Published:** 2007-02-07

**Authors:** Yuntao Wu, Margaret H Beddall, Jon W Marsh

**Affiliations:** 1 Section on Molecular Virology, Laboratory of Cellular and Molecular Regulation, NIMH, Bethesda, MD, 20892-4483, USA; 2 National Center for Biodefense and Infectious Diseases, Department of Molecular and Microbiology, George Mason University, Manassas, VA, 20110, USA

## Abstract

**Background:**

HIV-responsive expression vectors are all based on the HIV promoter, the long terminal repeat (LTR). While responsive to an early HIV protein, Tat, the LTR is also responsive to cellular activation states and to the local chromatin activity where the integration has occurred. This can result in high HIV-independent activity, and has restricted the use of LTR-based reporter vectors to cloned cells, where aberrantly high expressing (HIV-negative) cells can be eliminated. Enhancements in specificity would increase opportunities for expression vector use in detection of HIV as well as in experimental gene expression in HIV-infected cells.

**Results:**

We have constructed an expression vector that possesses, in addition to the Tat-responsive LTR, numerous HIV DNA sequences that include the Rev-response element and HIV splicing sites that are efficiently used in human cells. It also contains a reading frame that is removed by cellular splicing activity in the absence of HIV Rev. The vector was incorporated into a lentiviral reporter virus, permitting detection of replicating HIV in living cell populations. The activity of the vector was measured by expression of green fluorescence protein (GFP) reporter and by PCR of reporter transcript following HIV infection. The vector displayed full HIV dependency.

**Conclusion:**

As with the earlier developed Tat-dependent expression vectors, the Rev system described here is an exploitation of an evolved HIV process. The inclusion of Rev-dependency renders the LTR-based expression vector highly dependent on the presence of replicating HIV. The application of this vector as reported here, an HIV-dependent reporter virus, offers a novel alternative approach to existing methods, *in situ *PCR or HIV antigen staining, to identify HIV-positive cells. The vector permits examination of living cells, can express any gene for basic or clinical experimentation, and as a pseudo-typed lentivirus has access to most cell types and tissues.

## Background

All HIV-dependent expression vectors in common use are based on the HIV long terminal repeat promoter (LTR). An early HIV gene product, Tat, increases the level of transcript that is initiated at the LTR. The placement of reporter genes downstream of the LTR results in a responsiveness to the synthesis of Tat, a measure of HIV replication. The earliest indicator lines made use of reporter enzymes, such as luciferase and β-galactosidase [1-4], permitting a direct measurement of reporter gene induction. Tat appears to increase HIV transcriptional activity by two mechanisms. The first identified Tat activity is not directed towards the proviral DNA promoter, but rather through direct association with the growing nascent RNA chain. Tat associates with a 5' RNA loop structure [5-7], the transactivation response element (TAR), to promote completion of the initiated transcript [8-10], an activity also defined as processivity or anti-termination. More recent work has provided evidence that Tat also stimulates assembly of transcription factors to the DNA promoter [11]; that is, Tat promotes initiation, as well as elongation [12]. However, the LTR as a promoter is inherently leaky. Following integration of the HIV DNA into the host chromatin, the LTR transcribes the early gene products Tat, Rev, and Nef. That is, there is a required basal level of transcription that is Tat-independent. In addition, the site of integration or insertion of the LTR-based expression vector can mediate high levels of transcriptional activity in some cells [13], leading to expression from the reporter LTR in the absence of HIV. For example, in the generation of HIV indicator cells with LTR-based reporters, it has been necessary to remove 25% or more of the stably transfected cells [4,14] since they generate reporter transcript in the absence of HIV. While high expression cells can be removed in the generation of reporter clones, this inherent leakiness prevents the use of viral vectors to deliver the LTR-based reporter construct to detect the presence of existing HIV-positive cells in a mixed population.

By eliminating this non-specific activity, that is, non-HIV induction of signal, from an HIV expression vector, a wider use of this convenient and efficient tool would be possible. In addition to Tat, HIV transcriptional activity is also affected by an early gene product, Rev. Rev binds to a 3' loop structure, the Rev response element (RRE), present in unspliced and singly-spliced HIV transcripts, to permit nuclear export and translation of these mRNAs [15-17]. This viral specific activity exploits an essential cellular process. The removal of non-coding regions of transcripts (introns) prior to translation is critical to all cells. Introns are operationally defined by the presence of strong splicing sites, and HIV exploits this cellular activity by including multiple splice sites with varying activity [18,19] to generate multiple coding regions within the same stretch of HIV RNA. The existence of these sites results in the generation of fully spliced transcripts in the early phase of HIV infection [20,21]. Once Rev is expressed, RRE-containing HIV transcripts can be delivered to the cytosolic translational machinery.

It is of interest that in HIV infection protein expression from singly or non-spliced HIV transcripts appears absolutely dependent on Rev expression [15,22,23]. This dependency is lost with seemingly minor modifications to HIV DNAs [24,25], and the earliest reported RRE-containing expression constructs [26,27] can display varied Rev-independent expression. These were designed to elucidate Rev function, and the focus was not to eliminate background signal. In this report, by inclusion of multiple components of the HIV genome, we have constructed a lentiviral expression vector that displays a full dependency on the presence of HIV. As it is silent in HIV-negative cells, it differs dramatically from LTR-based systems.

## Results

**Figure 1:**
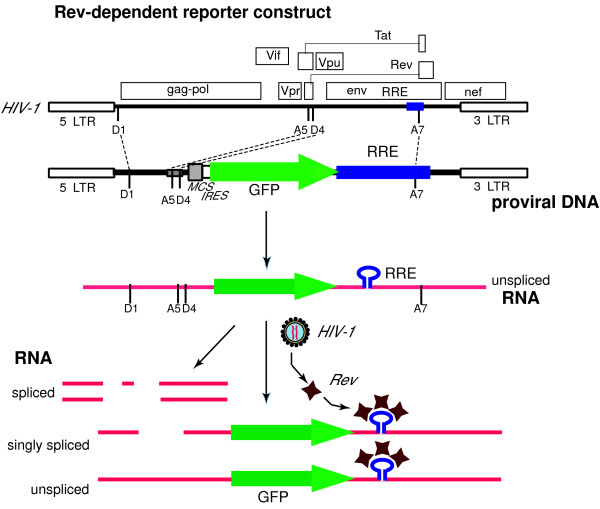
Rev-dependent reporter construct. A. Comparison of the HIV-1 genome and the Rev-dependent vector. The vector contains both LTRs along with the 5' end of the *gag *gene, splice donors and acceptors, and a portion of the *env *that includes the Rev response element. No intact HIV genes are present. As used in this report, the lentiviral vector contains an open reading frame encoding green fluorescent protein (GFP). B. In the absence of HIV infection the reporter provirus undergoes basal transcription generating a single message that is rapidly spliced, and results in the removal of the GFP reading frame. In the presence of HIV Rev, singly and non-spliced transcript are delivered to the cytosol, and the reporter gene is expressed.

To test the potential of Rev from HIV infection to control reporter gene expression in an HIV-dependent fashion, we constructed an HIV-like vector as shown in Fig. 1. Overall, we incorporated four separate segments of the HIV genome into the expression vector; however, no HIV gene is expressed from this construct. The 5' end of the vector consists of the HIV 5' LTR, the splice donor 1 site, D1, and a portion of the *gag *open reading frame that includes the packaging signal. The second HIV segment is from the *tat1/rev1 *exon that includes splice acceptor site 5, A5, and splice donor site 4, D4. The third segment of HIV DNA is from the *env *exon and encompasses the RRE, and the splice acceptor site 7, A7. The last segment includes the entire 3' LTR along with a small portion of the *nef *reading frame 5' to the LTR. In infectious HIV, the joining of splice donor 1 with splice acceptor site 5 (removal of D1/A5 intron) is utilized in transcripts for both Envelope and Nef proteins. In HIV NL4-3 infection of PBMC, the single spliced D1/A5 transcript represents 80% of all Env message [19]. Nef transcript requires a second splicing event, and the most common is removal of the segment between D4 and A7 [19]. The vector has the capacity to express two genes. A multiple-cloning site (MCS) for one of the expressed genes is immediately down-stream of the A5/D4 HIV splice sites; however, this cloning site has not been utilized in this report. This is followed by an internal ribosome entry site (IRES), which is upstream to the reporter gene used in this report, *green fluorescent protein *or GFP. The reporter gene is adjoined to the RRE-containing *env *exon segment. Together these segments compose the transfer vector. The full-length transcript generated from the construct possesses efficient HIV splice sites that mediate the removal of the reporter open reading frame (see Fig. 1). The reporter gene is only expressed from the RRE-containing unspliced or singly spliced transcripts, and thus requires the presence of Rev, a process that mimics HIV late gene expression. Co-transfection of this plasmid with an Env-coding plasmid and a packaging construct results in production of an infectious non-replicative lentiviral vector [28] capable of transferring the Rev-dependent reporter system into cells.

**Figure 2:**
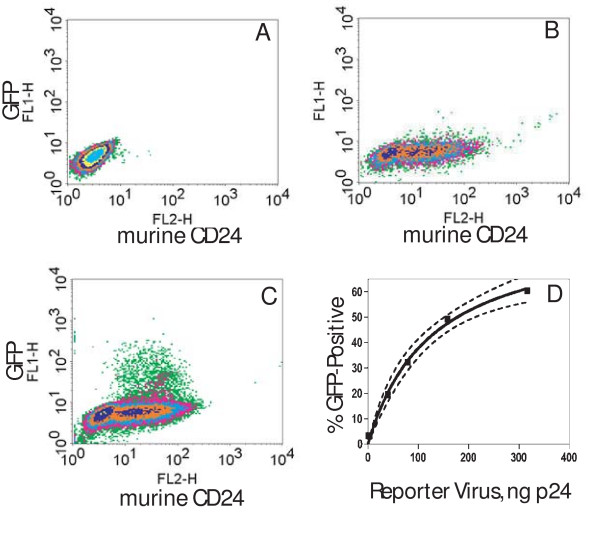
Specific expression of reporter by the Rev-dependent lentivirus in HIV infected T cells. CEM-SS cells were not infected (A) or were infected (B, C) with HIV-1 NL4-3.HSA.R+E-, an HIV-1 NL4-3 clone with the murine heat stable antigen (HSA; CD24) gene inserted into the *nef *gene. Both uninfected (A) and infected (C) cells were then transduced with the Rev-dependent reporter virus vNL-GFP-RRE(SA) (5 ng p24/10^6 ^cells; VSV-G envelope) or not transduced (B). At 72 hrs post transduction, cells were stained with R-phycoerythrin conjugated rat-anti-mouse CD24 antibody and analyzed by flow cytometer for CD24 and GPF expression. GFP was expressed only in HIV-1 infected (C), but not in HIV-1 uninfected (A) cells. Fifty thousand cells were examined in each run. In this run 61% of total cells were CD24-positive and of the CD24-positive population 17% were GFP-positive. Thresholds for CD24-positive and GFP-positive were set at a fluorescent intensity of 10 on the x- and y-axis respectively. Two independent analyses yielded the same result. D. Percent HIV-infected cells that express GFP versus reporter vector input. A concentrated preparation of reporter particles was used at varied dosages in HIV-infected CEM cells, as above. Flow cytometry studies yielded percent CD24-positive cells that became GFP-positive after 3 days (y-axis) versus reporter virus input (ng p24). Non-linear curve fitting (rectangular hyperbola) yielded R^2 ^= 99.4; maximum GFP-positive = 86.1 ± 6.3% (best-fit values ± std. error); K_D _= 128.1 ± 21.5 ng p24. Dashed lines are 95% confidence bands.

To permit a quantitative measurement of reporter gene induction from the lentiviral construct in response to HIV infection, we tested the vector in cells infected or not infected with HIV. CEM-SS cells, a human T cell line, were first infected with a modified, non-replicative NL4-3 HIV, where the gene for murine CD24 was inserted into the *nef *gene [29]. This NL4-3 strain possesses intact *tat *and *rev *genes, but lacks an *env *gene and therefore must be pseudo-typed (see Methods). Staining of cells for murine CD24 thus confirms the presence of HIV in a cell population (compare lack of staining in Fig. 2A to CD24-positive, HIV-infected cells in Fig. 2B). The resultant mixed populations of HIV-infected and non-infected CEM-SS cells were then transduced with a predetermined titer (see Methods) of the VSV-Env pseudo-typed Rev-dependent indicator lentiviral vector (Fig. 2A and 2C). In Fig. 2C, in order to determine whether a single reporter vector was adequately robust, we added one infectious indicator vector per five cells. If we assume that the infection is a Poisson process, then only two percent of the cells should contain two or more integrated indicator vectors. After 3 days, GFP was found expressed only in HIV-infected (CD24-positive) cells (Fig. 2C); HIV-free cells transduced with equivalent levels of indicator viral vector displayed no GFP signal (Fig. 2A). Consistent with the viral input, we found that approximately twenty percent of the CD24-positive cells (HIV^+^) were also GFP-positive (Fig. 2C; also see legend to Fig. 2). This finding demonstrates that a single infection of the reporter vector was adequate to detect the presence of HIV in the cell. To examine increased dosage of reporter virus, we utilized a second preparation of the lentiviral vector that had been concentrated by ultracentrifugation. As shown in Fig. 2D, the percent of HIV-infected cells (CD24-positive) that expressed GFP increased proportionally with increasing reporter viral vector input. The curve that fits this data is a rectangular hyperbola, defining this as a saturable process, and predicts that a maximum of 80–90% of the HIV-infected cells examined can be labeled by the Rev-dependent reporter virus (see legend Fig. 2 and Discussion).

**Figure 3:**
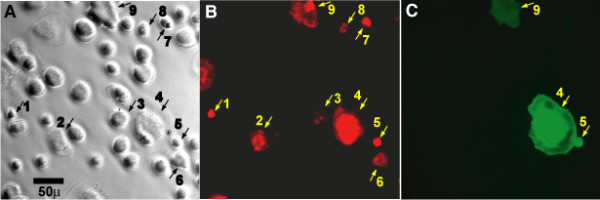
Specific expression of reporter by the Rev-dependent lentiviral vector in HIV-infected primary macrophages. M-CSF-treated monocyte-derived macrophages were infected or were not infected with HIV_AD8_, followed by Rev-dependent reporter virus vNL-GFP-RRE(SA). (A). Bright-field image of fixed cells with black bar representing 50 micrometers. Nuclei are prominent. Cells that were found to stain positive for HIV p24 are numbered in this image. B. Red fluorescence of identical field. All cells were stained for anti-p24 antibody, followed by a secondary red fluorescent antibody to detect productively infected cells. Intracellular red fluorescent foci identify productive infection; nine cells were identified in this field (B). We examined macrophage populations with four concentrations of HIV input (36, 72, 169, and 361 ng p24 HIV_AD8_/10^6 ^cells) followed by a constant input of reporter lentiviral vector (5 ng p24 vNL-GFP-RRE(SA)/10^6 ^cells; VSV-G envelope). Independent of the input of HIV, approximately one fifth of the p24-positive cells expressed GFP (23.0 ± 2.8%; mean ± SD; n = 4), as shown by green fluorescence in this same field (C). Magnification was identical on all three images. The reporter virus was demonstrated to function in macrophages from three donors.

Lentiviral vectors possess the capacity to infect differentiated macrophages, a known target cell for *in vivo *HIV replication. However, regulation of HIV LTR transcriptional activity in macrophages, compared to T cells, involves a different set of cellular factors [30,31]. We thus examined the capacity of the lentiviral expression vector to respond to HIV infection of macrophages. Human primary monocyte-derived macrophages were first infected with the CCR5-utilizing HIV-1_AD8 _followed 24 hours later by addition of the reporter lentiviral vector under conditions similar to the above CEM cell experiment. After 5 days the cells were fixed and stained for the presence of intracellular HIV p24 (Fig. 3). HIV-infected macrophages, stained for anti-p24, display cytosolic, perinuclear areas of high intensity due to accumulated viral particles, along with formation of large cells [32,33]. High intensity focal staining with anti-p24 (red fluorescence) was seen in multiple cells (see Fig. 3B, cells 1–9). Staining of cells in non-infected control populations was absent for both anti-p24 and GFP (data not shown), and GFP was expressed only in cells that were p24-positive (compare Fig. 3B and 3C). Of 308 cells examined here, 97 were p24-positive, and 22 of these were also GFP-positive. Under the conditions used in this experiment, and consistent with the viral input, approximately one fifth of the p24-positive macrophages displayed GFP production. As with the CEM experiment (Fig. 2), this finding suggests that a single infecting indicator viral vector in macrophages is capable of reporting the presence of HIV.

**Figure 4:**
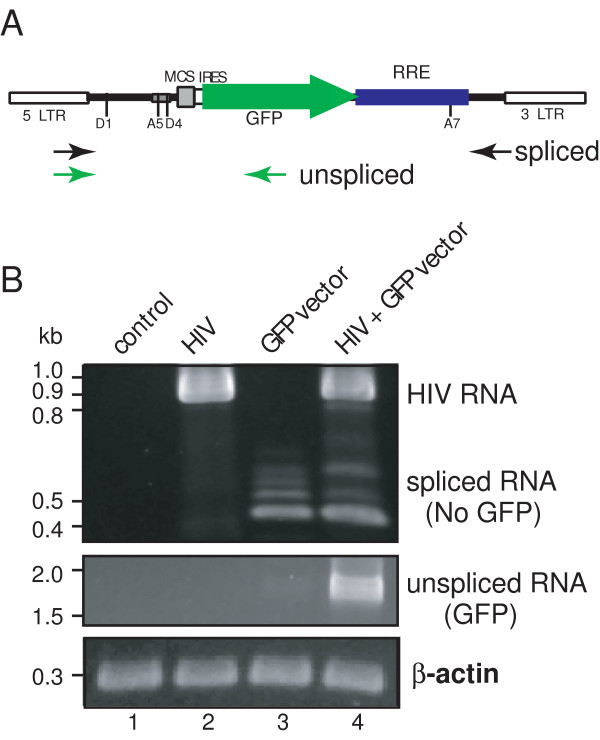
Reverse transcriptase PCR analyses of gene expression from lentiviral vector NL-GFP-RRE-(SA) in response to HIV-1 infection. (A) A diagram of PCR primers used. Two sets of primers were used to detect the spliced (black arrows) and unspliced (green arrows) transcripts by reverse transcriptase PCR (RT-PCR) as described in Methods. The mRNA molecules from infected and uninfected cells were extracted at day 3 after lentiviral vector infection and analyzed by RT-PCR for the spliced transcripts and unspliced transcripts as shown in (B). (B) CEM-SS cells were either uninfected (lane 1, control), infected with HIV alone (lane 2, HIV, VSV-G pseudo-typed, 564 ng p24 per million cells), infected with NL-GFP-RRE-(SA) alone (lane 3, 1764 ng p24 virion per million cells), or infected with HIV first, then NL-GPF-RRE(SA) 24 hours later (lane 4). The cellular *β-actin *transcript was also amplified to ensure that comparable numbers of cells were used for the analyses.

The Rev-dependent system outlined in Fig. 1 operates under the assumption that in the absence of HIV there is elimination, through splicing, of GFP-encoding transcript. Upon Rev expression, the non-spliced RRE-containing GFP transcript would be rescued. To test this hypothesis, we performed RT-PCR on control and HIV-infected CEM-SS cells that were secondarily exposed to the reporter GFP-encoding viral vector at high input levels (see Fig. 4 legend). High levels of reporter vector were used to challenge the cell's capacity to remove GFP-encoding non-spliced transcript in the absence of HIV, that is, to remove false-positive reporter message. We used two sets of primers that amplified the spliced or non-spliced transcripts from the lentiviral construct (Fig. 4A), and compared spliced to unspliced reporter transcript in four cell populations: uninfected cells, cells infected with HIV only, cells infected with reporter virus only, and cells infected with both virus. Note that the vectors used to detect spliced transcript will also recognize HIV transcripts. As shown in Fig. 4B, HIV-specific transcript, consistent with the size of fully spliced transcript amplified with these primers (0.94 kb), appeared in the two cell populations infected with HIV (lanes 2 and 4). In cells exposed to the reporter vector but lacking HIV, we observed basal levels of transcription from the LTR promoter of the reporter vector (lane 3). This transcriptional activity represents HIV-independent LTR-mediated signal (the leakiness seen with LTR-based systems); however, with this Rev-dependent system the transcripts were all spliced (lane 3; spliced RNA vs unspliced RNA) and thus lacked the capacity to generate the GFP reporter. Following exposure to the reporter viral vector, the generation of a non-spliced, reporter-encoding message (seen in lane 4) was dependent on the presence of HIV infection. These data confirmed that expression of GFP-encoding transcript was HIV-dependent, and that the dependency corresponded to the prevention of transcript splicing, consistent with Rev activity.

## Discussion

The Rev-dependent expression vector displayed highly specific HIV-dependent expression. The application tested here, as a lentiviral reporter for HIV detection, permitted us to address two major concerns: non-specific (HIV-independent) noise and inadequate specific (HIV-dependent) signal. The first concern is based on knowledge that expression from an integrated LTR vector can be affected by the local chromatin activity, resulting in HIV- or Tat-independent high expression [13]. Of course, this is what prevents utilization of LTR-based reporter viruses. With the establishment of LTR-based indicator cells, it's just a matter of removing cells with aberrantly high background reporter expression [4,14]. This problem appeared to be absent in cells transduced with the Rev-dependent vector system, since expression of GFP in both a T cell line and a primary macrophage population occurred only in HIV-positive cells. Furthermore, we found that GFP-encoding transcript was detected by RT-PCR only in HIV-infected populations. Secondly, we wished to know whether this vector, potentially as a single integrant and with the added stringency of Rev-dependence, would generate an adequately robust response. To address this point we examined reporter function under conditions that maximized single reporter integrants. It does assume that infections follow a Poisson function, a central tenet to most virus infection studies. At an input where 80% of the cells do not become infected (approximately one infectious particle per five cells), less than 2% of the cells should possess more than one reporter provirus. Expression of GFP reporter from the HIV-positive cells closely approximated the input reporter viral vector level (Fig. 2 and 3), suggesting that undetected expression of integrated vectors is not prominent. Note, however, that expression of the reporter gene is achieved in cells that are also highly supportive of transcriptional activity from the HIV provirus, that is, LTR activity is supported. Both HIV and the reporter vector use the LTR promoter.

We also examined high doses of reporter viral vector, where a majority of the cells are infected with the Rev-dependent reporter vector. Under these conditions we did not see intact reporter (unspliced) transcript or protein in HIV-negative cells. The nature of the curve that fits the reporter vector detection of HIV-infected cells (Fig. 2D) suggests that the rate-limiting step towards reporter expression follows Michaelis-Menten kinetics. This is characteristic of a single binding-site event, but at this time we are unable to define this rate-limiting process, such as the viral particle binding to the cellular receptor. Additional mechanisms leading to this non-linear function would include the potentially equivalent susceptibility of increasing numbers of infected cells to additional infection, as characterized by a Poisson distribution. Concentration of this viral particle by centrifugation permitted this examination of high vector input, but it also diminished the infectivity of the viral particles. This data also predicts that most of the HIV-infected cells will be susceptible to detection by the reporter virus. Of course, in mixed cell populations or tissues there will be variable susceptibilities to infection by the reporter virus as presently used. The inability to infect all cells by the reporter virus is perhaps the most limiting aspect of this application of the Rev-dependent vector.

In this report we have utilized the term *Rev-dependence *to distinguish this construct from the existing LTR-based Tat-responsive HIV reporter systems; however, with the intact LTR, we would speculate that this Rev-dependent vector should maintain responsiveness to Tat expression as well. This dual dependency is likely to contribute to the higher stringency seen. In this document, we have characterized this Rev-dependent vector in only one application (viral reporter vector), an application that is challenging and not feasible with the LTR-based reporters. At the same time we view this application as supplementary to existing methods to test cells for the presence of HIV replication. These include the staining or probing of HIV proteins and nucleic acids in fixed cells, and may include amplification with PCR [34-36]. We have also incorporated the vector into continuous cell lines (data not shown). As introduced into a transformed T cell, we find that expression from this Rev-dependent vector is insensitive to cellular activation states, thus maintaining a negative background in the absence of HIV, but has enhanced sensitivity to HIV infection, relative to many of the existing non-T cell indicator cell lines. Although this report has been limited to the expression of reporter genes, the construct's incorporation into a lentivirus and the specificity acquired should also permit its use as an experimental agent to express any gene in HIV-infected cells.

## Conclusion

The inclusion of Rev-dependent processing to an LTR-based vector has resulted in a highly specific HIV-dependent expression system.

## Methods

### Viruses and cells

Viruses and cells were obtained through the NIH AIDS Research and Reference Reagent Program: pNL4-3.HSA.R+E- from Dr. Nathaniel Landau [29]; CEM-SS from Dr. Peter L. Nara [37]; J1.1 cells from Dr. Thomas Folks [38]. HIV-1_AD8 _is a macrophage-tropic molecular clone [39]. Macrophages were derived from peripheral blood monocytes of healthy donors from the Department of Transfusion Medicine at the National Institutes of Health. Cells were cultured in RPMI media supplemented with 10% fetal bovine serum (Hyclone) and recombinant human macrophage colony stimulating factor (rH-MCSF) (R & D Systems) at 10 ng/ml. Media was changed every 48 hours for ten days.

### DNA constructs

pCMVΔR8.2 and pMD.G have been described previously [28]. pNL-GFP-RRE was constructed by complete deletion of all HIV ORFs of pNL4-3 [40] by replacing the 8.1 kb BssHII-BlpI fragment of the HIV-1 genomes with an insert containing the GFP ORF and the HIV-1 Rev-responsive element (RRE) including the HIV-1 sequence immediately following the BssHII site and the first 336 nucleotides of the gag ORF (the gag reading frame was disrupted by a frame shift mutation at the ClaI site by blunt end ligation), the GFP ORF was derived from pIRES-hrGFP-1a (Stratagene) by PCR amplification (5' CTCGAAATTAACCCTCACTAAAGG 3'; 5'ATCGTGTACGGCCGAATTGGGTACACTTACCTG 3'), and the fragment containing RRE (corresponding to position 7612 to 8469 of the HIV-1NL4-3 genome). pNL-GFP-RRE-(SA) was constructed by insertion of a PCR fragment into the NotI-SmaI site of pNL-GFP-RRE, in front of the GFP ORF. The insert carrying the HIV-1 A5 splicing acceptor and D4 donor was amplified by primers: 5' ATAAGAATGCGGCCGCATCTCCTATGGCAGGAAG 3'; 5' AATCACCCGGGTGCTACTACTAATGCTACTATTGC 3'. The sequence of pNL-GFP-RRE-(SA) has been deposited in GenBank (accession number EF408805).

### Virus generation

Stocks of the HIV-1_NL4-3 _and HIV-1_AD8 _were prepared by transfection of HeLa cells with cloned proviral DNA. HIV-1 based lentiviral vectors carrying reporter genes were made by cotransfection of DNA constructs as follows: 2 × 10^6 ^293T cells were cotransfected with 10 ug of pNL-GFP-RRE-SA, 7.5 ug of pCMVΔR8.2, and 2.5 ug of pMD.G using the calcium phosphate method (Promega). Viral particles were harvested 2 days after cotransfection and filtered through a 0.45 um filter and stored at -80°. One preparation was concentrated by ultracentrifugation [41]. The titer (TCID_50_) of the lentiviral indicator vNL-GFP-RRE(SA) virus preparation was estimated by serial dilution into activated (TNF-treated) HIV-positive J1.1 Jurkat cells [38] following the method of Reed and Muench [42].

### RT-PCR analysis

Total cellular poly(A^+^) mRNA was purified from cells by MicroPoly(A)Pure mRNA isolation kit (Ambion) as recommended by the manufacturer. Reverse transcription was accomplished using the RETROscript First-Strand Synthesis Kit (Ambion) with random decamers as the first-strand primers. Following cDNA synthesis, PCR was carried out using primer 5'TAATCGGCCGAACAGGGACTTGAAAGCGAAAG3' and 5'CAGGCACAAGCAGCATTGTTAG 3' to amplify spliced lentiviral transcripts, and primer 5' TAATCGGCCGAACAGGGACTTGAAAGCGAAAG3' and 5'ATCGTGTACGGCCGAATTGGGTACACTTACCTG3' to amplify non-spliced GFP transcripts. The PCR condition was: 1 × PCR buffer, 125 uM dNTPs, 1.5 mM Mg^2+^, 50 pmol of each primer, 1 U SuperTaq Plus (Ambion) in 50 ul, with 30 cycles of 20 sec at 94°, and 180 sec at 68°. One fifth of the product was analyzed on 2% agarose gel. Cellular *β-actin *transcripts were amplified using QuantumRNA β-actin Internal Standards (Ambion) with similar conditions as above except using 20 pmol of each actin primer and runs at 20 sec at 94°, 30 sec at 55°, and 40 sec at 68°.

### Reporter virus detection of HIV infection

CEM-SS cells, infected with VSV pseudo-typed HIV-1 NL4-3.HSA.R+E- [29], were enriched by biotin conjugated rat-anti-mouse CD24 antibody and streptavidin conjugated magnetic beads and further cultured for two weeks to remove the beads. The enriched population was then infected with the Rev-dependent lentiviral vector, vNL-GFP-RRE(SA). At 72 hrs post transduction, cells were stained with R-phycoerythrin conjugated rat-anti-mouse CD24 antibody and analyzed by flow cytometer for CD24 and GPF expression. Curve fitting and statistical analysis were achieved with GraphPad Prism software.

Monocyte-derived macrophages in six well plates were infected with HIV_AD8 _(36 to 360 ng p24) in 500 ul of RPMI for 3 hours, then washed and returned to 2 ml RPMI. The following day, 5 ng p24 of pNL-GFP-RRE(SA) was applied to the macrophages. At 5 days post infection, cells were intracellularly stained on ice. Briefly, cells were rinsed once with Hank's buffer followed by treatment with Cytofix/Cytoperm (Becton Dickinson) for 20 min, then 2 ml of cold methanol for 15 min. After washing with Permeabilization/Wash buffer (Becton Dickinson), cells were stained with anti-p24 HIV antibody 183.H12-5C (1:250) [43-45] for 90 min, followed by washing and staining with Alexa Fluor-568 goat anti-mouse IgG (Invitrogen) (1:250) for 30 minutes.

### Fluorescent microscopy of monocyte-derived macrophages

The GFP-expressing and anti-p24 stained cells were photographed using a Leica DMIRB/E inverted research microscope attached to a Orca DCAM ER camera (Hamamtsu), using an XF102.2 filter (Omega Optical Inc.) for the Alexa Fluor-568 (p24) detection and a GFP filter (Leica) for the GFP expressing cells. P24 expression and GFP expression were captured with similar exposures, typically 1000 milliseconds. Files were saved as Tiffs and color was added on ImageJ software version 1.33u (Rasband, W., NIH-public domain). Dimensions were determined through a stage micrometer (Electron Microscopy Sciences).

### Flow cytometry

Infected or uninfected cells were re-suspended into 100 ul Hank's staining buffer (Hank's buffer plus 0.1% bovine serum albumin, 0.1% sodium azide, and 25 mM HEPES, pH 7.2) for staining with antibodies at concentrations recommended by manufactures. Following staining for 30 min on ice, cells were either washed with Hank's buffer or, in the case of HIV-infected cells, fixed with 500 ul of Cytofix/Cytoperm (Becton Dickenson) for 20 min on ice for flow cytometry analysis on a FACScan analyzer (Becton Dickenson). The murine CD24 antibody was from Southern Biotechnology.

## Competing interests

A patent application for the vector has been submitted by NIMH with YW and JWM.

## Authors' contributions

YW, MB, and JM performed experiments, performed data analysis, contributed to experimental design, and contributed to writing this paper.
